# LAI-2 adjunctive treatment for type I Bipolar patients with comorbid Obsessive Compulsive Disorder: preliminary data from a real-world multi-centric Italian clinical experience

**DOI:** 10.1192/j.eurpsy.2023.1491

**Published:** 2023-07-19

**Authors:** V. Martiadis, E. Pessina, A. Martini, F. Raffone, P. Giunnelli, D. De Berardis

**Affiliations:** 1 Department of Mental Health, ASL Napoli 1 Centro, Napoli; 2 Department of Mental Health, ASL Cuneo 2, Cuneo; 3 Department of Mental Health, Asl Teramo, Teramo, Italy

## Abstract

**Introduction:**

Comorbidity with Obsessive Compulsive Disorder (OCD) in patients with bipolar disorder (BD) affects from 10 to 20% of the clinical samples considered. The pharmacological treatment of these patients emphasizes the clinical issue of the use of serotonergic anti-obsessive agents, which may increase the risk of manic/mixed episodes or may accelerate a rapid cycle course. In some cases, the addition of a second stabilizer drug results in improvement in both mood disorder and comorbid obsessive psychopathology. Although off-label, the use of II generation long-acting injectable antipsychotics (LAI-2) in type I BD is widespread in clinical practice but data regarding their efficacy in improving obsessive symptoms of the eventual comorbid disorder are still lacking.

**Objectives:**

The aim of this open-label naturalistic study was to evaluate the efficacy and safety of adjunctive treatment with LAI-2 monthly paliperidone palmitate (PP1M-LAI) and monthly aripiprazole (ARI-LAI) in 24 bipolar type I BD patients with OCD comorbidity, in a real-world clinical setting of 3 outpatient services located in the 3 macro-areas of Northern, Central and Southern Italy.

**Methods:**

Twenty-four patients diagnosed with type I BD and comorbid OCD were recruited and observed over a 24-week period after the add-on of PP1M-LAI or ARI-LAI to stabilizing therapy. Psychopathology assessment was performed by means of Yale-Brown Obsessive Compulsive Scale (YBOCS), Hamilton Depression Rating Scale (HDRS), Brief Psychiatric Rating Scale (BPRS), Young Mania Rating Scale (YMRS), Hamilton Anxiety Rating Scale (HARS). The mean PP1M-LAI dosage was 117.8 mg/month while that of ARI-LAI was 400 mg/month.

**Results:**

At the end of the observation period, all patients who completed the study demonstrated a consisten reduction in obsessive symptoms while maintaining effective mood stability in the absence of signs of hypomanic/manic change (YBOCS mean reduction 24,5 to 16,2, GLM r.m. p<0.001; HDRS mean reduction 19 to 10, GLM r.m. p<0.001; YMRS mean reduction from 23,2 to 6,3, GLM r.m. p<0.001). The relatively small number of patients recruited did not allow to detect significant differences in the performance of PP1M and ARI-LAI. Overall tolerability was good for both treatments, in line with the tolerability profiles of each drug.

**Conclusions:**

While considering the limitations of the relatively small sample and the open-label design, the results of this study indicate that the two LAI-2, PP1M and ARI-LA,I can be considered an effective and well-tolerated treatment in type I BD patients with OCD comorbidity, confirming efficacy in mood stabilization and reducing obsessive symptoms. Further studies on larger samples will be needed to confirm these preliminary findings and to detect any performance difference between the two antipsychotics.

**Disclosure of Interest:**

None Declared

